# High-throughput RNA sequencing reveals differences between the transcriptomes of the five spore forms of *Puccinia striiformis* f. sp. *tritici*, the wheat stripe rust pathogen

**DOI:** 10.1007/s44154-023-00107-z

**Published:** 2023-07-31

**Authors:** Gangming Zhan, Jia Guo, Yuan Tian, Fan Ji, Xingxuan Bai, Jing Zhao, Jun Guo, Zhensheng Kang

**Affiliations:** 1 State Key Laboratory of Crop Stress Biology for Arid Areas and College of Plant Protection, Northwest A&F University, Yangling, Shaanxi 712100 P.R. China; 2 School of Pharmacy, Guizhou University of Traditional Chinese Medicine, Guiyang, Guizhou 550025 P.R. China

**Keywords:** *Puccinia striiformis* f. sp. *tritici*, Transcriptome, Gene expression, Rust life cycle, Spore stages

## Abstract

The devastating wheat stripe (yellow) rust pathogen, *Puccinia striiformis* f. sp. *tritici* (*Pst*), is a macrocyclic and heteroecious fungus. *Pst* produces urediniospores and teliospores on its primary host, wheat, and pycniospores and aeciospores are produced on its alternate hosts, barberry (*Berberis* spp.) or mahonia (*Mahonia* spp.). Basidiospores are developed from teliospores and infect alternate hosts. These five spore forms play distinct roles in *Pst* infection, disease development, and fungal survival, etc. However, the specific genes and mechanisms underlying these functional differences are largely unknown. In this study, we performed, for the first time in rust fungi, the deep RNA sequencing to examine the transcriptomic shift among all five *Pst* spore forms. Among a total of 29,591 identified transcripts, 951 were specifically expressed in basidiospores, whereas 920, 761, 266, and 110 were specific for teliospores, pycniospores, aeciospores, and urediniospores, respectively. Additionally, transcriptomes of sexual spores, namely pycniospores and basidiospores, showed significant differences from those of asexual spores (urediniospores, teliospores, and aeciospores), and transcriptomes of urediniospores and aeciospores were more similar to each other than to the three other spore forms. Especially, the basidiospores and pycniospores which infected the berberis shows wide differences in the cell wall degrading-enzymes and mating and pheromone response genes. Besides, we also found that there are 6234 differential expressed genes between the urediniospores and pycniospores, while only have 3 genes have alternative splicing enents, suggesting that differential genes expression may make more contribution than AS. This comprehensive transcriptome profiling can substantially improve our understanding of the developmental biology of the wheat stripe rust fungus.

**Supplementary Information:**

The online version contains supplementary material available at 10.1007/s44154-023-00107-z.

## Introduction

*Puccinia striiformis* f. sp. *tritici* (*Pst*) is an obligate biotrophic fungus that causes serious stripe rust disease in wheat (Chen 2020). *Pst* has a complex life cycle comprising five different spore stages, uredinial, telial, basidial, pycnial and aecial, on two botanically distinct plant hosts, the primary host of wheat (*Triticum aestivum*), and the alternate hosts of barberry (*Berberis* spp.) or mahonia (*Mahonia* spp.) (Jin et al. 2010; Wang and Chen 2013).

Urediniospores are dikaryotic and infect the primary host wheat through stomata to accomplish the asexual cycle. Asexual cycles can repeat several times during one growing season and the resulting urediniospores can be carried over long distances by wind, potentially leading to severe wheat epidemics (Zhao et al. 2016). During the later stages of wheat growth or when weather conditions are not suitable for urediniospore infection and production, *Pst* produces teliospores in wheat (Zhao et al. 2016). Teliospores can tolerate much higher temperatures than urediniospores, and they can overwinter on decaying wheat leaves. A teliospore contains two cells, each of which initially contains two nuclei and becomes diploid after karyogamy. Meiosis takes place before germination of mature teliospores (Hacquard et al. 2013). Teliospores are important for the overwintering of many rust fungi, and they are largely responsible for sexual recombination, mediating the formation of new races (Anikster and Wahl 1979). After karyogamy and meiosis, each cell of the teliospore produces four haploid basidiospores. Basidiospores infect barberry (*Berberis* spp.) or mahonia (*Mahonia* spp.) through direct penetration of epidermal cells, then produce pycnia on the abaxial side of the leaves (Zhao et al. 2016). Pycniospores released from pycnia function as gametes and fuse with receptive hyphae in pycnia of an opposite mating type. After fertilization, dikaryotic mycelia grows within the host tissue and produces aecia, which contain dikaryotic aeciospores, on the adaxial side of the leaves. Aeciospores then infect wheat to complete the life cycle. Even though we have gained wealth of knowledge on the biological features and functions of these five spore forms for the rust fungi, the molecular mechanisms underlaying their biology are still poorly understood.

The genomic and transcriptomic analyses provide opportunities to accelerate our understanding the biology of the rust fungi, for example the identification of virulence-related genes and their expression patterns during the wheat-*Pst* interactions (Cantu et al. 2013; Xia et al. 2017, 2022, 2020; Tian et al. 2016, 2017; Wang et al. 2018; Peng et al. 2021). But most of the studies have primarily focused on the uredinial stage, encompassing urediniospores, infectious hyphae, and haustoria within host plant tissue (Ling et al. 2007; Yin et al. 2009; Huang et al. 2011; Zheng et al. 2013; Xia et al. 2018a, 2018b). For example, Garnica et al. (2013) compared the transcriptomes of germinated urediniospores and haustoria and proposed that urediniospores rely mainly on stored energy reserves for growth and development, whereas haustoria take up host nutrients and generate massive energy to support biosynthetic pathways and the production of new urediniospores. Dobon et al. (2016) found by RNA-seq that a sequential, temporally coordinated activation and suppression of immune-response regulator expression was responsible for the outcome of *Pst*-wheat interactions. Overall, these studies aimed to identify differentially expressed genes and effectors related to virulence by examining wheat-*Pst* interactions at the asexual stages.

In-depth transcriptome studies of sexual stages, in addition to asexual stages, will be helpful to paving the way to dissect the regulatory mechanism underlying the host adaptation in heteroecious rust fungi (Duplessis et al. 2021). In fact, only a few studies have identified genes involved in the sexual stages of rust fungi. Xu et al. (2011) sequenced expressed sequence tags (ESTs) to characterize gene expression patterns among pycniospores, aeciospores, teliospores, and germinated urediniospores of *Puccinia triticina*, the wheat leaf rust pathogen. In addition, Hacquard et al. (2013) compared expression profiles of *Melampsora larici-populina* at the telial and uredinial stages, and identified genes associated with overwintering as well as numerous lytic enzymes related to plant cell wall degradation. Lorrain et al. (2017) compared the expression patterns of *M. larici-populina* among basidia, pycnia, and aecia in poplar and larch, and found that the majority of genes encoding secreted proteins were expressed in both hosts. However, the transition of spore forms and the underlying expression patterns need to be investigated in the *Pst*.

To the best of our knowledge, there are no published reports on how gene expression in the spore forms directly relates to the sexual cycle of *Pst*. Therefore in the present study, we performed, for the first time in the rust fungi, the transcriptomic analyses for all five spore forms of *Pst*, including urediniospores, teliospores, basidiospores, pycniospores, and aeciospores, and conducted pairwise comparisons of gene expression among them. We identified spore-specific transcripts in each stage and found the alternative splicing (AS) events were more extensive between urediniospores and pycniospores. These genes may be involved in specific developmental processes, and the results of this study may elucidate the complex life cycle of *Pst*.

## Results

### Morphological characteristics of the five spore stages

**Fig. 1:**
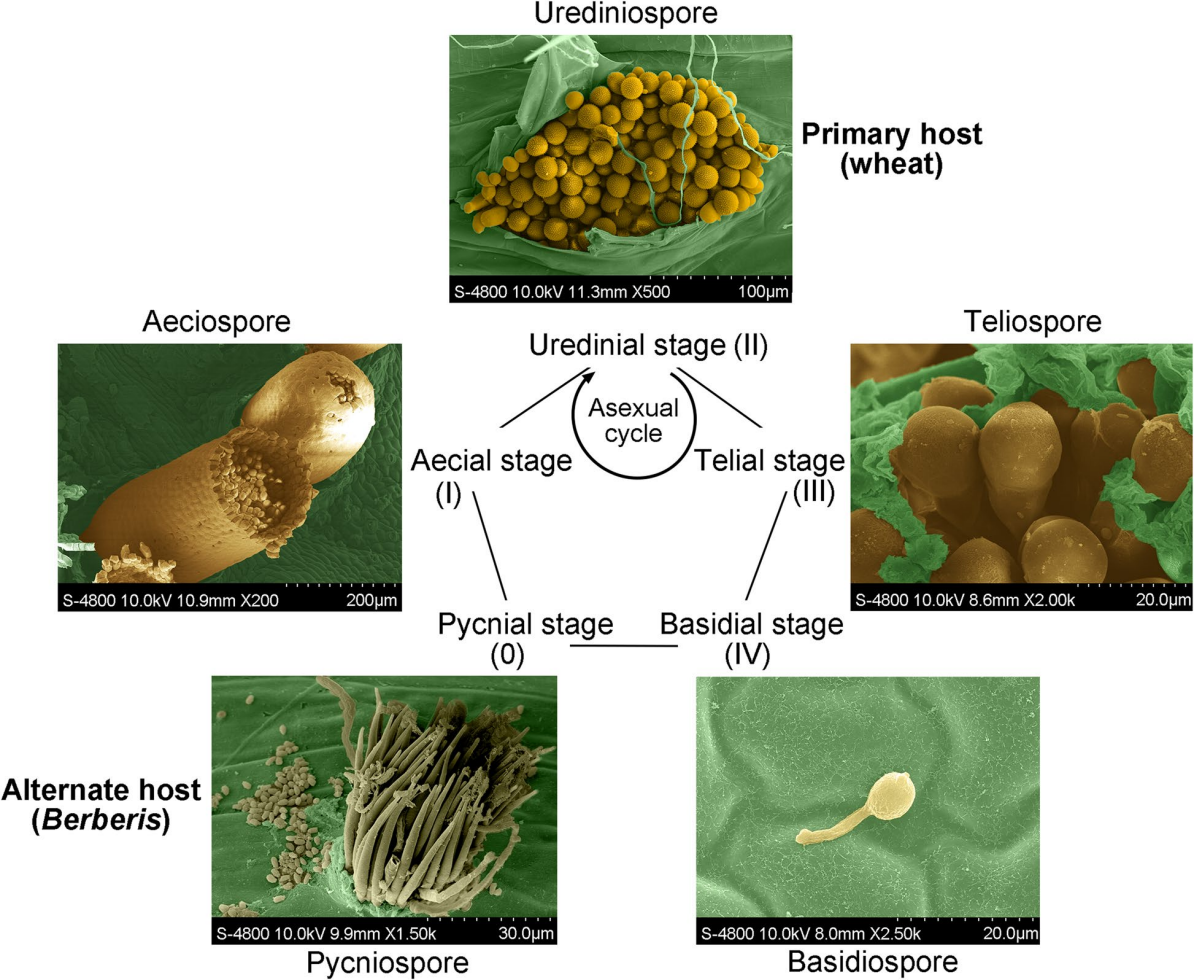
Morphological characteristics of the five spores and life cycle. Five type of spores and simplified life cycle of *Puccinia striiformis* f. sp. *tritici*. The colors were marked depend on the natural phenotype by photoshop

*Pst* have the most complex life cycle with a macrocyclic life style, containing five different types of spores, and require two botanically unrelated hosts. To exhibit the morphological characteristics of the five different types of spores, the spores were collected and observed by scanning electron microscope. The colors were marked depend on the natural phenotype by photoshop (Fig. 1). The urediniospores (yellow-orange), the mainly spore type of disease epidemics, can reinfect wheat (primary host) to cause multiple reinfections within a cropping season. The teliospores (dark yellowish-brown) are produced in wheat at the late growth stage with impoverished environment conditions. The teliospores geminated and give rise to basidiospores (light yellow, almost hyaline). The basidiospores can infect berberis (alternate host) to produce pycnia and pycniospores (hyaline) with paraphyses and trichogyne. The sexual life cycle is completed with the mating type and trichogyne, and producing the aecia on berberis leaves. The aeciospores (yellow-orange) are released from the broken aecia and infect wheat to produce urediniospores (Zhao and Kang 2023).

### Transcriptomic analyses of the five spore stages

**Fig. 2:**
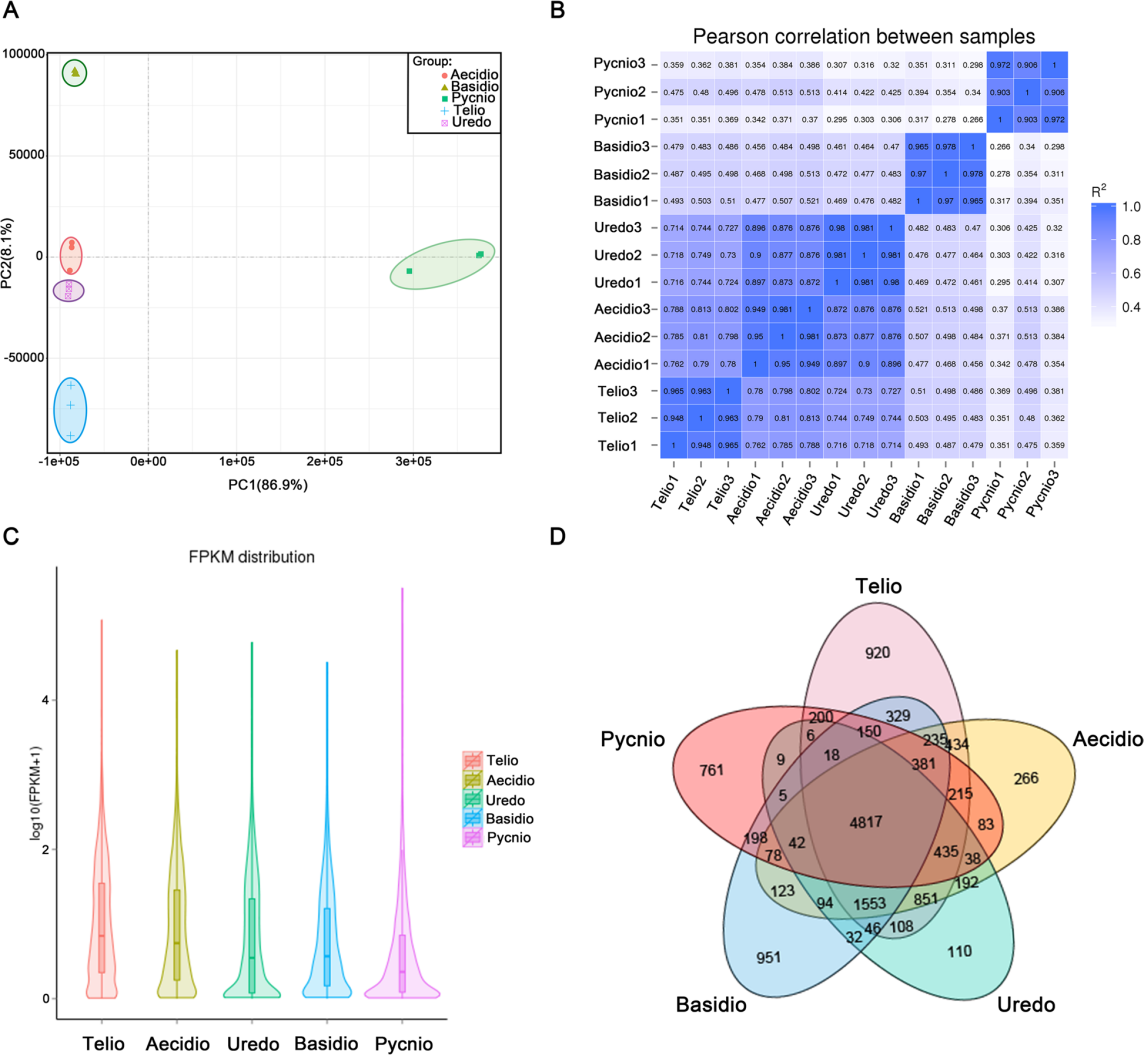
Expression analysis of five spore stages. **A** Principal component analysis (PCA) of urediniospores, teliospores, basidiospores, pycniospores, and aecidiospores measured using fragments per kilobase of exon per million fragments mapped (FPKM). The PCA plot places biological replicates along the two PC axes, explaining 86.9% (x-axis) and 8.1% (y-axis) of the variance within samples. **B** Pearson correlation of samples. **C** the expression level of the genes FPKM in different samples. **D** Venn diagram showing the number of genes expressed in urediniospores (green), teliospores (purple), basidiospores (blue), pycniospores (orange), and aeciospores (yellow)

In order to screen different transcriptome of five different types of *Pst* spores, and gain a comprehensive understanding of the gene expression dynamics, five different types of *Pst* spores cDNA libraries were sequenced by Illumina HiSeq platform. On average, 43.5 million RNA-seq reads were generated per sample, of which 37.4 million were successfully mapped to the CY32 reference genome. Total mapping ratios ranged from 85.62% to 91.36%, except for two teliospore samples with only 60.10% and 71.73%, respectively (Table S1). Replicates within the same sample type were highly similar based on principal component analysis (PCA) and Pearson correlation analysis (Fig. 2A, B), indicating a high reproducibility of the RNA-seq data. The transcriptomes of urediniospores and aeciospores were more closely related to each other than to the other three spore forms (Fig. 2A).

**Fig. 3:**
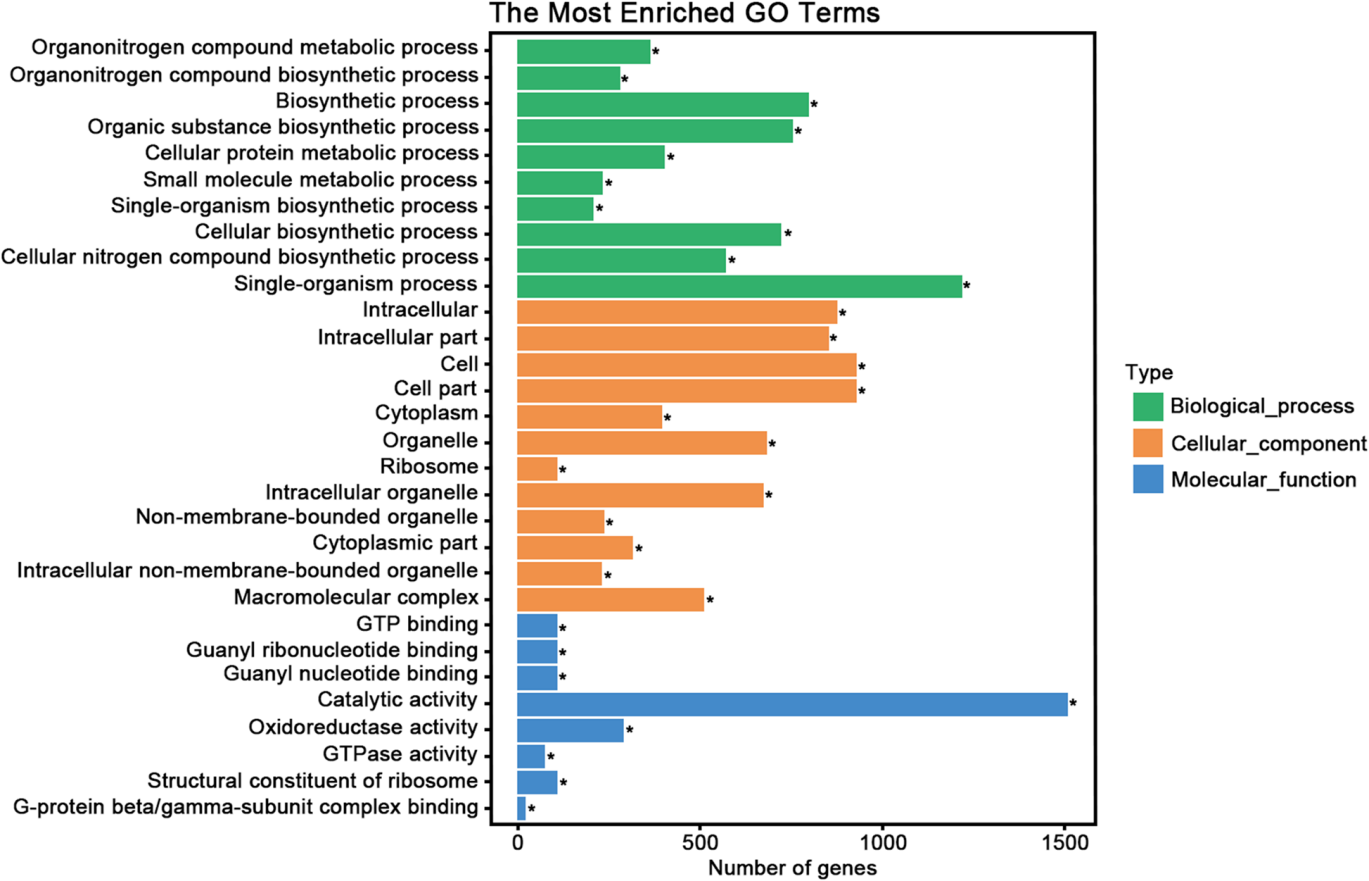
GO enrichment analysis of genes expressed in all spore stages. Thirty of the most significantly enriched GO terms of genes expressed in all spore stages. * indicates significant enrichment

HTSeq v0.9.1 was used to count the reads numbers mapped to each gene. And then FPKM.of each gene was calculated based on the length of the gene and reads count mapped to this gene. Based on a threshold value of 1.0 for average FPKM, a total of 29,591 transcripts were detected in all 15 RNA-seq datasets, and among them were 2,759 sequences which were not annotated in the reported CYR32 reference genome. The expression level of the genes FPKM were exhibited no significant difference between samples (Fig. 2C). Of the 29,591 transcripts, 4,817 were expressed in all spore stages (Fig. 2D). GO enrichment analysis determined that the functions of these commonly expressed genes were mainly related to metabolism, biosynthesis, and transport (Fig. 3, Table S2). The number of transcripts detected in teliospores, aeciospores, basidiospores, urediniospores, and pycniospores with a FPKM > 1 were 10,698, 9,837, 9,052, 8,356, and 7,436, respectively. Moreover, 951 (10.5%), 920 (8.5%), 761 (10.2%), 266 (2.7%), and 110 (1.3%) transcripts were detected to be specifically expressed in basidiospores, teliospores, pycniospores, aeciospores, and urediniospores, respectively (Fig. 2D). Many specifically expressed genes encoded proteins with unknown functions, observed in 85.7%, 80.9%, 67.7%, 65.0%, and 54.5% of pycniospores, basidiospores, teliospores, aeciospores, and urediniospores, respectively. GO term enrichment analysis for genes specifically expressed in pyciniospores and basidiospores are shown in Table S3. For the other three spore stages, no GO term enrichments were found.

### Differentially expressed genes (DEGs) among pairwise comparisons

The respective transcriptomes were pairwise compared to better understand the difference in gene expression among the five spore forms. Differential expression analysis of was performed using the DESeq R package (1.18.0). In total, 12,653 transcripts were significantly differentially expressed in at least one pairwise comparison (padj < 0.05) (Table S4). The fewest DEGs were found between urediniospores and aeciospores (Table 1). The highest number (8,347) of DEGs was detected between urediniospores and basidiospores, followed by the number of DEGs between basidiospores and pycniospores. The summary of DEGs in all pairwise comparisons is given in Table 1.

**Table 1:** Pairwise comparison of the number of genes differentially expressed in different spore stages of *Puccinia striiformis* f. sp. *tritici*

	**No. of genes**		
**Pair of spore stages**	**Differential expressed (*P* < 0.05)**	**Up-regulated**	**Down-regulated**
Teliospore vs urediospore	5,971	3,298	2,673
Teliospore vs basidiospore	7,932	3,712	4,220
Teliospore vs pycniospore	5,077	2,129	2,948
Teliospore vs aeciospore	3,127	1,351	1,776
Aeciospore vs urediospore	2,781	1,867	914
Aeciospore vs basidiospore	7,238	3,339	3,899
Aeciospore vs pycniospore	4,827	1,995	2,832
urediospore vs basidiospore	8,347	3,366	4,981
urediospore vs pycniospore	6,234	2,399	3,835
Basidiospore vs pycniospore	6,942	3,398	3,544

### Clustering of DEGs

**Fig. 4:**
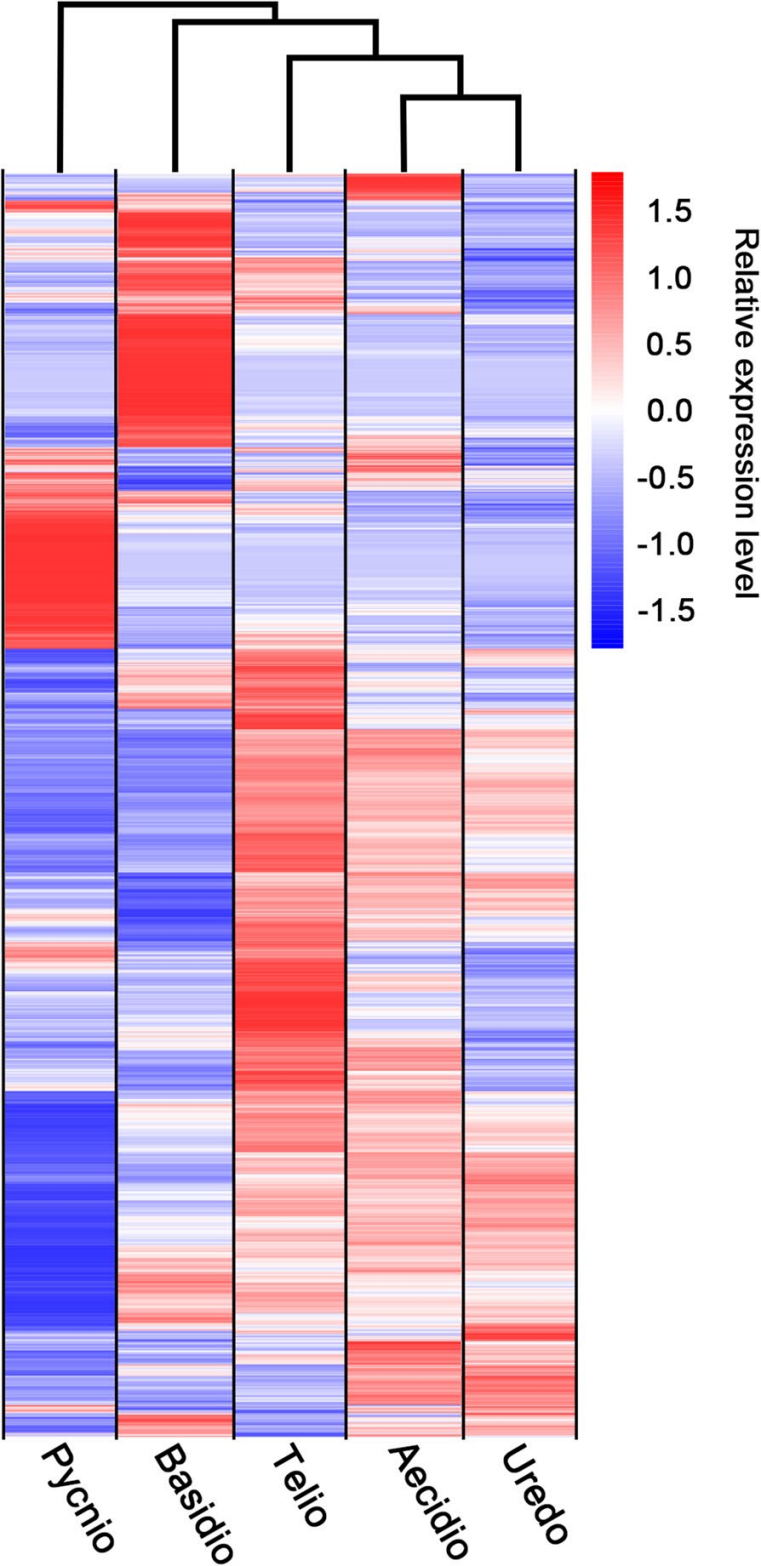
Hierarchical clustering analysis of gene expression profiles. FPKM heatmaps of differentially expressed genes (DEGs) identified in all pairwise comparisons with FDR < 0.05. Over-expression (red) and under-expression (blue) are shown relative to the expression (log10(FPKM + 1)) measured across all five spore forms

**Fig. 5:**
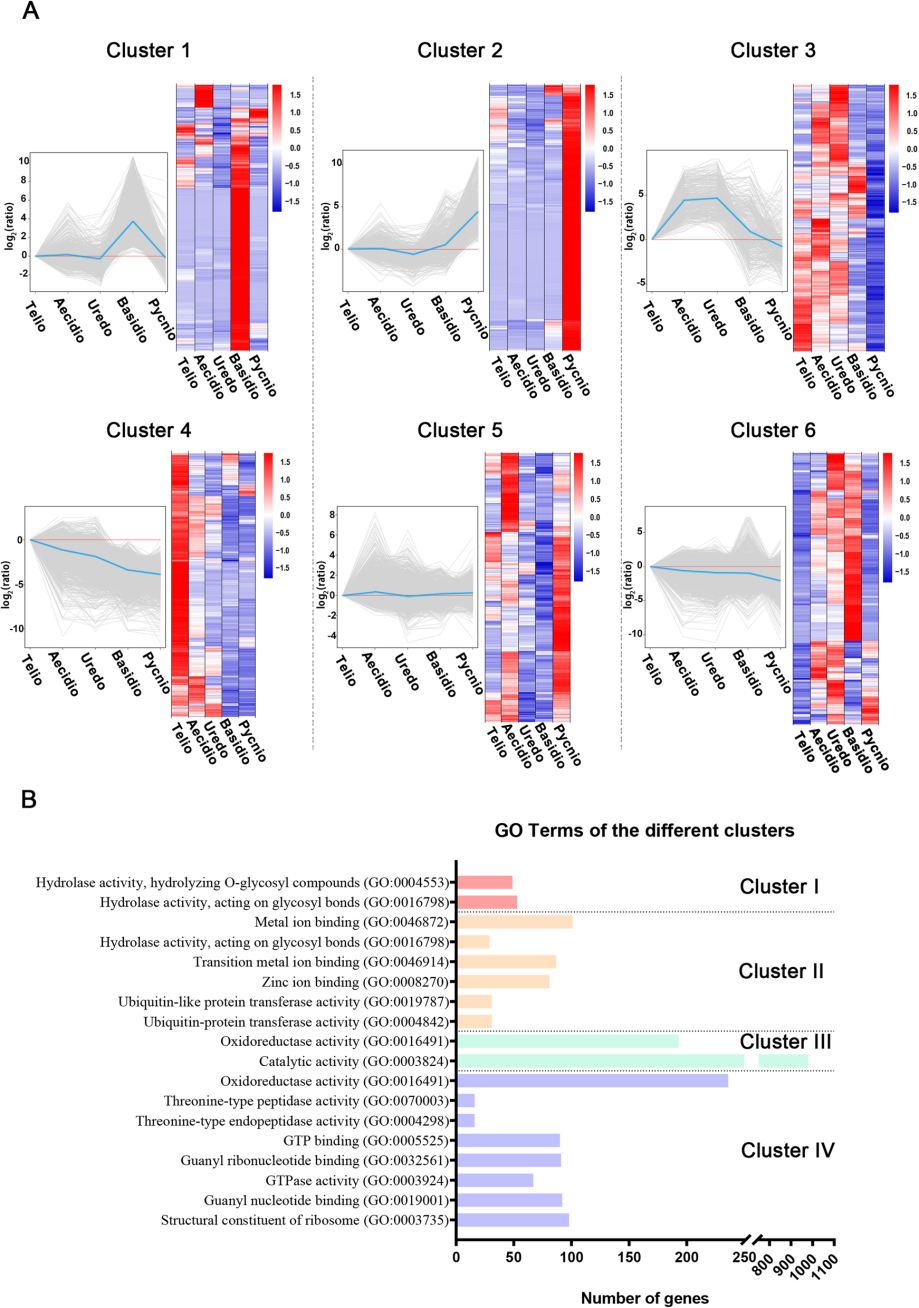
Hierarchical clustering analysis of gene expression profiles of six sub-clusters. **A** gene expression profiles of six sub-clusters. **B** enriched Go terms

Hierarchical clustering analysis was performed for the 12,653 DEGs detected in at least one pairwise comparison between spore forms. The DEGs grouped into six clusters. Transcripts from urediniospores were more similar to those from aeciospores and teliospores, and while all were distantly related to those from pycniospores and basidiospores, the latter two were most closely related (Fig. 4). To characterize the functional differences of different clusters, DEGs were analyzed by GO term enrichment to explore their relevant molecular functions (Table S5). Cluster 1 mainly consisted of genes significantly up-regulated in basidiospores (Fig. 5), including genes involved in the carbohydrate metabolic process and hydrolase activity. Cluster 2, which mainly contained genes up-regulated in pycniospores, was enriched with ubiquitin transferase and hydrolase activity. Cluster 3 contained mainly down-regulated genes in pycniospores and was enriched in catalytic and oxidoreductase activities. Cluster 4, including a large number of genes up-regulated in teliospores, was enriched for structural constituents of ribosome, guanyl nucleotide binding, and GTPase activity. Cluster 5 contained some genes up-regulated in pycniospores, aeciospores, and teliospores. Cluster 6 included genes up-regulated in urediniospores, aeciospores, and basidiospores. No GO functions were enriched for genes in clusters 5 and 6.

### Functional classification of DEGs

To further understand the differences in transcriptomes among the different spore forms of *Pst*, DEGs between urediniospores and other spore forms were subjected to GO term enrichment analyses. Compared to urediniospores, genes involved in cellular components and molecular functions related to translation, including ribosome (GO:0005840) and structural constituent of ribosome (GO:0003735), were more active in teliospores, aeciospores, and pycniospores. Genes up-regulated in basidiospores were enriched in hydrolase activity, such as acting on glycosyl bonds (GO:0016798) and hydrolyzing O-glycosyl compounds (GO:0004553). These two GO terms were also found in pycniospores. Biological processes, including sexual reproduction (GO:0019953), spermatogenesis (GO:0007283), male gamete generation (GO:0048232), and gamete generation (GO:0007276), were enriched in pycniospores. Genes involved in ubiquitination were also enriched in pycniospores. Detailed GO functional annotation of DEGs between urediniospores and other spore forms are provided in Table S6.

### Differentially expressed carotenoid biosynthesis-related genes explained pigmentation of *Pst* spores

The five *Pst* spores differ in color. Teliospores are dark yellowish-brown, basidiospores and pyciniospores are hyaline, and aeciospores and urediniospores are yellow-orange. The yellow-orange color is due to carotenoid pigments, whereas the dark yellowish-brown color of teliospores may result from an accumulation of diphenol/urishiol (multi-copper) oxidase or laccase (Hennessy & Sackston 1972; Xu, et al. 2011). In the present study, we checked the variation of gene expression related to carotenoid biosynthesis and diphenol/urishiol (multi-copper) oxidase or laccase. Genes up-regulated in aeciospores were enriched in carotenoid metabolic (GO:0016116) and biosynthetic processes (GO:0016117) when compared to enrichment in basidiospores. Three of the ten genes enriched in carotenoid metabolic and biosynthetic processes were up-regulated in urediniospores and aeciospores compared to results in the other spore stages. Two genes encoding (multi-copper) oxidase or laccase (*PSTCY32_09115*, *Novel01958*) showed high transcript accumulation in teliospores compared to that in urediniospores and basidiospores.

### DEGs between urediniospores and basidiospores revealed different penetration mechanisms of *Pst* spores

Heteroecious rust fungi infect two taxonomically different host species. *Pst* urediniospores infect wheat, whereas basidiospores infect barberry. Transcriptomic differences of the two spore forms may explain the different types of infection processes used on the two hosts (penetration through stomata on wheat and direct penetration of epidermal cells on barberry). In the present study, 1,523 genes were identified with a minimum of ten-fold change in expression levels in basidiospores compared to urediniospores (Table S7). These DEGs were analyzed through functional annotation based on a BLASTP search against the Swissprot database. Among the 1,523 genes, 1,411 were up-regulated in basidiospores, including genes encoding plant epidermis and plant cell wall degrading enzymes such as cutinase (6 genes), cellobiohydrolase (6), pectinesterase (2), pectin lyase (2), and glucanase (5), while of the 112 genes that were highly expressed in urediniospores, there was only one cutinase and one glucosidase (Table 2). DEGs found in basidiospores compared to urediniospores also included some kinase-coding genes (Table 2).

**Table 2:** Genes of *Puccinia striiformis* f. sp. *tritici* differentially expressed in basidiospores and urediospores

		**Spore stage**	
**Definition**	**Gene ID**	**Basidio**	**Uredio**
*Cell wall degrading enzymes*			
Cutinase	PSTCY32_23882	Up	Down
	PSTCY32_23883	Up	Down
	PSTCY32_23884	Up	Down
	PSTCY32_00598	Up	Down
	PSTCY32_20087	Up	Down
	PSTCY32_00600	Up	Down
Cellobiohydrolase	PSTCY32_20078	Up	Down
	PSTCY32_06175	Up	Down
	PSTCY32_06177	Up	Down
	PSTCY32_06174	Up	Down
	Novel02413	Up	Down
	Novel02414	Up	Down
Pectinesterase	PSTCY32_06064	Up	Down
	PSTCY32_04373	Up	Down
Pectin lyase	PSTCY32_12745	Up	Down
	PSTCY32_23675	Up	Down
Glucanase	Novel02147	Up	Down
	PSTCY32_25679	Up	Down
	PSTCY32_25686	Up	Down
	PSTCY32_19986	Up	Down
	Novel02147	Up	Down
Kinase		Up	Down
Serine/threonine-protein kinase	PSTCY32_20947	Up	Down
	PSTCY32_21791	Up	Down
	PSTCY32_07193	Up	Down
	PSTCY32_21268	Up	Down
Calcium/calmodulin-dependent protein kinase	PSTCY32_16143	Up	Down
Mitogen-activated protein kinase	PSTCY32_06131	Up	Down
	PSTCY32_06134	Up	Down
*Cell wall degrading enzymes*			
Cutinase	PSTCY32_05662	Down	Up
Glucosidase	PSTCY32_23279	Down	Up
**Number**		**29 up, 2 down**	**29 down, 2 up**

### Highly induced genes in pycniospores were related to sexual reproduction

Basidiospores and pycniospores are produced after meiosis from teliospores. Some genes related to mating and the pheromone response pathways were found to be highly expressed (fold change > 10) in basidiospores and pycniospores when compared to those in urediniospores (Tables S7 and S8). In basidiospores, pheromone receptor gene STE3.3 (*PSTCY32_07086*), pheromone-regulated membrane protein 10 gene Prm10 (*PSTCY32_21004*), and scaffold protein gene Scd2 (*PSTCY32_14093*) were highly expressed. Pheromone receptor genes STE3.2 (*PSTCY32_04929*) and STE3.3 (*PSTCY32_07086*), sexual differentiation process protein gene *isp4* (*PSTCY32_12519*, *PSTCY32_25336*), and plasma membrane fusion protein gene Prm1 (*PSTCY32_17608*) were highly expressed in pycniospores.

### Karyogamy and meiosis-related genes were highly expressed in teliospores

Karyogamy and meiosis are crucial cellular processes that take place in teliospores (Hacquard et al. 2013). In the present study, several genes related to karyogamy and meiosis were found to be significantly expressed in teliospores. For example, a gene encoding nuclear fusion protein Kar5 (*PSTCY32_04109*) and a gene encoding meiosis-specific serine/threonine-protein kinase Mek1 (*PSTCY32_01818*) were highly expressed in teliospores with 11.19-fold and 15.73-fold changes compared with the expression levels in urediniospores, respectively (Table S9). Teliospores are also known as dormant and oversummering and overwintering spores. One aquaporin gene (*AQY1*: *PSTCY32_18461*) (fold change 10.16 compared with urediniospores) potentially related to such a process was found to be abundant in teliospores. Four heat shock protein genes (Hsp16: *PSTCY32_19847*, *PSTCY32_21361*, *PSTCY32_23508*, *PSTCY32_05903*) (fold change > 10 compared with urediniospores) were also highly expressed in teliospores.

### Alternative splicing (AS) events among different spores

**Fig. 6:**
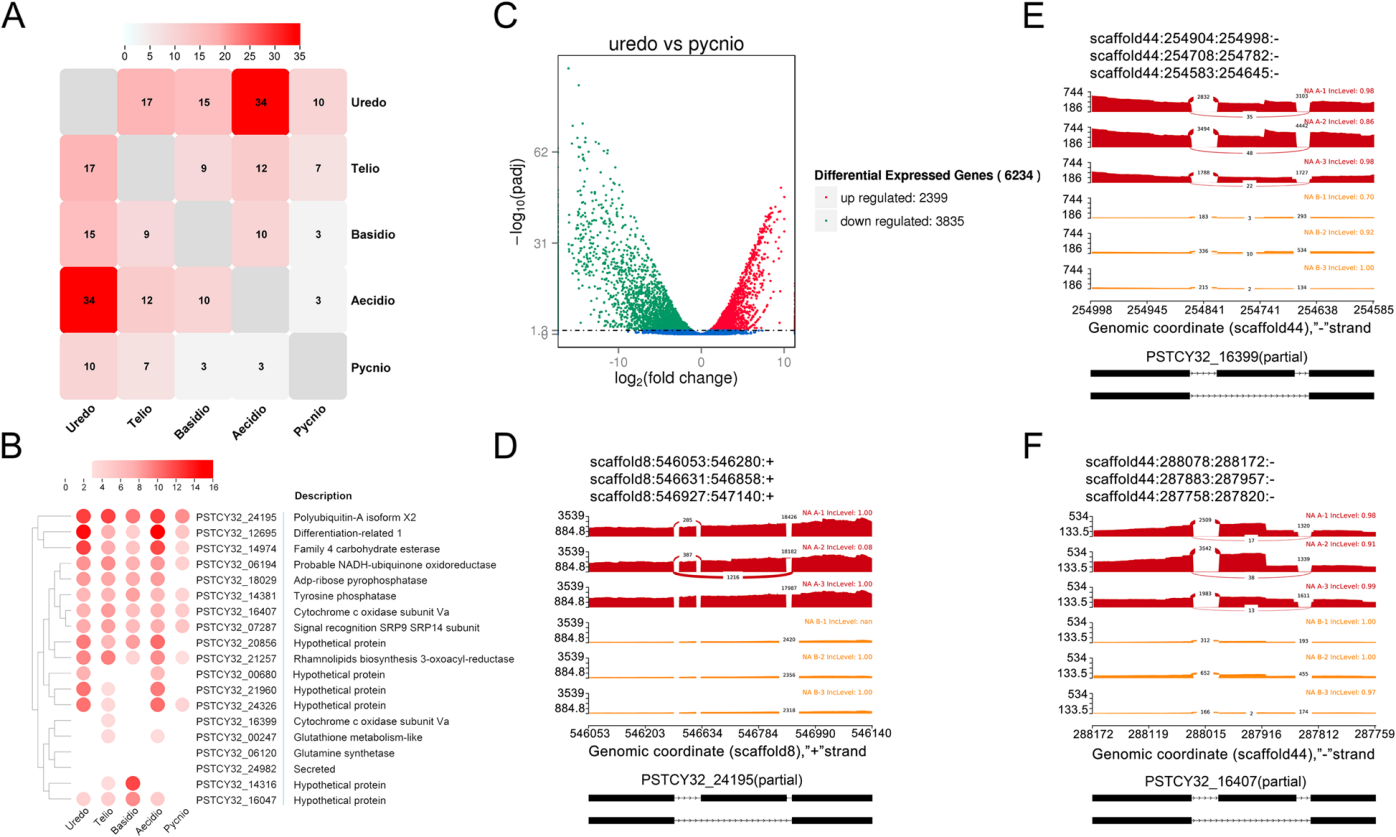
AS event identification in 19 genes. **A** AS events among five spore types in pairs. **B** FPKM heatmaps of DEGs with AS events identified in all pairwise comparisons with FDR < 0.05. Over-expression (red) and under-expression (blue) are shown relative to the expression (log2(FPKM + 1)) measured across all five spore forms. **C** differential expressed genes between the urediniospores and pycniospores. **D**, **E**, and **F** The AS event plot of PSTCY32_24195, PSTCY32_16407, and PSTCY32_16399 in the urediniospores vs pycniospores group, respectively

AS is a ubiquitous mechanism for regulating gene expression with multiple mRNAs from one gene. AS events were identified among the five kinds of spores in pairs. Seventeen AS events (13 Skipped exon (SE) and 4 Mutually exclusive exon (MXE)) were identified between urediniospores and teliospores, 15 AS events (10 SE and 5 MXE) were identified between urediniospores and basidiospores, 34 AS events (23 SE and 11 MXE) were identified between urediniospores and aeciospores, and 10 AS events (7 SE and 3 MXE) were identified between urediniospores and pycniospores. Nine AS events (8 SE and 1 MXE) were identified between basidiospores and teliospores, 3 AS events (3 SE) were identified between basidiospores and pycniospores, and 10 AS events (7 SE and 3 MXE) were identified between basidiospores and teliospores. Three AS events (2 SE and 1 MXE) were identified between aeciospores and pycniospores and 12 AS events (9 SE and 3 MXE) were identified between aeciospores and teliospores. Lastly, 7 AS events (6 SE and 1 MXE) were identified between pycniospores and teliospores (Fig. 6A, Table S10). All AS events were identified in only 19 genes with high expressions except for PSTCY32_06120 and PSTCY32_24982 (Fig. 6B). To compare the number of differentials expressed genes with the number of AS events between urediniospores (asexual stage) and pycniospores (sexual stage), the differentials expressed genes was shown in Fig. 6C. There are 6234 genes with 2399 up regulated and 3835 down regulated. The polyubiquitin-A isoform X2 protein (PSTCY32_24195) and two cytochrome c oxidase subunit Va (PSTCY32_16399 and PSTCY32_16407) exhibited SE in the urediniospores vs pycniospores group (Fig. 6D, E, and F). PSTCY32_24195 has the SE eventfrom 546,630 to 546,858, which lacked the C-terminal of Iron Transport-associated domain (Fig. 6D). PSTCY32_16399 and PSTCY32_16407 have the SE event from 254,707 to 254,782 and from 287,882 to 287,957 respectively, which were similar occurred in C-terminal and did not affect the conserved domain (Fig. 6E, and F).

## Discussion

The wheat stripe rust fungus is heteroecious and throughout its life cycle, five different spore forms, urediniospores, teliospores, basidiospores, pycniospores, and aeciospores, play various crucial roles. Knowledge of the development and function of these different spores is fundamental for developing novel disease control strategies. In this study, we performed high-throughput RNA sequencing with the five types of spores to understand their functional genomics differences.

In total, 4,817 genes were found expressed in all five types of spores. In general, these genes were related to biosynthesis, metabolism, and transportation (Fig. 2), indicating that they were conserved and essential for the basic activities and entire life cycle of *Pst*. Basidiospores had the largest number of stage-specifically expressed genes, followed by teliospores, pycniospores, aeciospores, and urediniospores. This pattern was similar to a previous study of *P. triticina* without basidiospores (Xu et al. 2011).

The yellow-orange rust color of urediniospores and aeciospores can be attributed to carotenoid pigments accumulating in lipid droplets within the cytoplasm as well as pigments associated with the cell wall (Hennessy and Sackston 1972). Basidiospores and pycniospores, however, are hyaline. Unlike results in basidiospores, genes up-regulated in aeciospores were enriched in both carotenoid metabolic and biosynthetic processes. Among these genes, three were up-regulated in all colored spores (urediniospores, teliospores, and aeciospores) and not in the hyaline spores (basidiospores and pycniospores). This may explain the different colors of these two groups of spores. Teliospores are survival structures and are melanized for stress conditions (Xu et al. 2011). Laccase, which oxidizes different phenols and diamines, has been implicated in the production of melanin (Xu et al. 2011). Two genes encoding laccase (*PSTCY32_09115*, *Novel01958*) were highly expressed in teliospores. Genes with similar functions have also been identified in an EST library of *P. triticina* teliospores and the transcriptome of teliospores from the poplar rust fungus *M. larici-populina* (Hacquard et al. 2013; Xu, et al. 2011). These genes could be involved in the biosynthesis of melanin.

More genes related to plant epidermis and cell wall degradation, including cutinase, cellobiohydrolase, pectinesterase, pectate lyase, and glucanase, were found among the DEGs in basidiospores when compared with DEGs in urediniospores. Different kinds of cell wall-degrading enzymes were discovered in basidiospores and urediniospores. Basidiospores showed 21 highly expressed genes that were encoding enzymes related to cellulose, hemicellulose, pectin, and cutin degradation. However, only two genes encoding cellulase and cutinase were found expressed in urediniospores. These enzymes are responsible for the degradation of plant cell walls to facilitate the attachment, invasion, and colonization of the host plant (Schafer, 1994). These findings suggested that different cell wall-degrading enzymes were utilized during the penetration of different hosts. Basidiospores are short-lived, surviving only a few hours under ideal conditions (Roelfs et al. 1982). They germinate rapidly by producing a germ tube with a terminal appressorium from which an infection peg develops and directly penetrates the plant epidermal cell wall. However, urediniospores can remain alive for months under appropriate conditions and infect wheat leaves through stomata. Therefore, genes related to cell wall-degrading enzymes may be induced earlier and to a higher extent in basidiospores than in urediniospores. Basidiospores also had a higher accumulation of some kinase genes than urediniospores. These kinases may regulate the pathogenesis processes of basidiospores. The cell wall degrading-enzymes and kinases induced in spore stages may be prepared in advance of infection.

Basidiospores are produced after meiosis from teliospores, and at this stage, genes involved in mating and pheromone response pathways are required. Indeed, pheromone receptor gene *STE3.3* (*PSTCY32_07086*) was highly expressed in basidiospores. In Basidiomycota, the sexual cycle typically requires cell–cell fusion governed by both pheromone and pheromone receptor (Raudaskoski and Kothe 2010). After pheromones are recognized by specific receptors expressed on the surface of cells of opposite mating types, they activate the expression of mating-specific genes in mitogen-activated protein kinase (MAPK) pathways (Merlini et al. 2013). The establishment of cell polarity and mating responses in the fission yeast *Schizosaccharomyces pombe* is regulated by a signaling complex that involves the scaffold protein Scd2 (Chang et al. 1999). In the present study, genes involved in fusion pathway components were found highly expressed in basidiospores. This was exemplified by the highly expressed genes encoding pheromone-regulated membrane protein Prm10 (*PSTCY32_21004*) and scaffold protein Scd2 (*PSTCY32_14093*) in basidiospores.

Pycniospores function as gametes and fuse with receptive hyphae that functions as the opposite gamete. In our GO analysis, GO terms of sexual reproduction (GO:0019953), spermatogenesis (GO:0007283), male gamete generation (GO:0048232), and gamete generation (GO:0007276) were all found enriched in pycniospores. Mating type-specific genes were also highly expressed in pycniospores. Two pheromone receptors (STE3.2 and STE3.3), which acted as pheromone receptors on the surface of cells to bind pheromones of the opposite mating type, were highly induced in pycniospores. Furthermore, plasma membrane fusion protein Prm1, a pheromone-regulated membrane glycoprotein involved in the plasma membrane fusion event of *Saccharomyces cerevisiae* mating (Olmo and Grote 2010), was also highly expressed in pycniospores. Another gene related to the sexual differentiation process, *isp4*, as well as its homologs, was highly expressed in *Pst* pycniospores, which is similar to the identification of these genes in pycniospores of *P. triticina* (Xu et al. 2011).

Teliospores are vital for the overwintering of most cereal rust fungi. Aquaporins, which have been found in Bacteria, Archaea, and Eukaryotes, serve to enhance cellular tolerance against rapid freezing (Tanghe et al. 2006) by reducing intracellular ice crystal formation through the rapid, osmotically driven efflux of water during the freezing process (Tanghe et al. 2002). In the present study, *AQY1* was found highly induced in teliospores, suggesting an important role in the survival of teliospores during winter. One aquaporin, (*Mlp-26257*), orthologous to *AQY1* in *M. larici-populina*, is specifically expressed in telia (Hacquard et al. 2013). In our study, four genes encoding heat shock proteins (Hsp16) were highly induced in teliospores. Heat shock treatment was found to increase the expression of the *hsp16* gene in *Schizosaccharomyces pombe* (Taricani et al. 2001). Teliospores are usually formed during high temperatures. Heat shock protein genes expressed highly in teliospores may relate to heat resistance, helping *Pst* teliospores to survive summer.

Karyogamy is an essential step for the intermixing of parental genetic information during sexual reproduction in dikaryotic fungi (Fu and Heitman 2017). In *S. cerevisiae*, Kar5 localizes to both inner and outer nuclear membranes at the spindle pole body and coordinates the outer and inner nuclear membrane, facilitating the inner nuclear membrane fusion step during karyogamy (Rogers and Rose 2015). In the present study, *Kar5* was up-regulated in teliospores, which may be related to its requirement for membrane fusion during karyogamy, similar to its *M. larici-populina* ortholog that is transiently induced and accumulated during the karyogamy process (Hacquard et al. 2013). *Mek1* is a meiosis-specific kinase in budding yeast which promotes recombination between homologous chromosomes by suppressing double-strand break repair between sister chromatids (Niu et al. 2007). High expression of *Mek1* was detected in teliospores, indicating the important role of *Mek1* during meiosis in teliospores.

AS can extend the diversity of mRNA and protein isoforms as well as change the expression of gene isoforms (Kim et al. 2014). Previous studies have reported the involvement of AS events in sex determination and gonadal differentiation in many species (Carreira-Rosario et al. 2016; Cesari et al. 2020; Gómez-Redondo et al. 2021; Yu et al. 2021). However, more detailed knowledge of AS remains limited. In this study, AS events were identified among pairs of the five kinds of spores. The total AS events was less than found in other species, and most were identified in the urediniospores vs aeciospores pairing, with only 34. In *Pst*, only 10 AS events were identified between the urediniospores (asexual stage) and pycniospores (sexual stage), including one polyubiquitin-A isoform X2 protein (PSTCY32_24195) and two cytochrome c oxidase subunit Va (PSTCY32_16407 and PSTCY32_16399) with SE events. Meanwhile, there are 6234 differential expressed genes between the urediniospores and pycniospores. These results suggested that the AS events were unimportant for the asexual stage transfer to sexual stage and for the generation of different spores.

## Conclusions

In the present study, we firstly performed deep RNA sequencing of the five types of *Pst* spores: urediniospores, teliospores, basidiospores, pycniospores, and aeciospores. Our data illustrates the gene expression patterns of different spore forms and characterizes the functional features of DEGs identified between different spore forms. Especially, the basidiospores and pycniospores which infected the berberis shows wide differences in the cell wall degrading-enzymes and mating and pheromone response genes. Besides, we also found that there are 6234 differential expressed genes between the urediniospores and pycniospores, while only have 3 genes have alternative splicing enents, suggesting that differential genes expression may make more contribution than AS. Our results will enhance the understanding of the developmental biology of different spore forms and lay a foundation for further study on the functions of genes and gene products induced at each stage.

## Methods

### Sample preparation and RNA extraction

PL17-7, a single-spore isolate of *Pst* obtained from a stripe rust leaf sample collected in 2017 in Gansu province, China, was used in this study, as well as susceptible wheat cultivar MX169. To begin propagation, wheat seedlings at the two-leaf stage were inoculated with urediniospores and incubated in a dew chamber at 10 °C for 24 h in darkness, then grown at 16 °C with a 16/8 h light/dark cycle to produce urediniospores. These urediniospores were harvested into a glass tube and stored at -80 °C for further analysis. To induce teliospore formation, MX169 seedlings bearing a high density of uredia were transferred to a greenhouse with temperatures of 25 °C in daylight and 16 °C at night, under a 16/8 h light/dark cycle. One month after inoculation, teliospores were harvested by scratching telia from senescent leaves with a sterile scalpel, after which the samples were snap-frozen in liquid nitrogen and stored at -80 °C. Wheat leaf segments bearing teliospores were kept in petri dishes to induce basidiospore formation using the following method described by Zhao et al. (2013). When abundant basidiospores were observed under the microscope, petri dishes containing wheat leaf segments were flipped over and gently shaken so that basidiospores would fall onto the inner surface of the petri dish lids. RNAlater (Qiagen, Hilden, Germany) was added to the petri dishes to collect basidiospores. This collection step was repeated three times every six hours. To inoculate barberry with basidiospores, wheat leaf segments bearing germinated teliospores were placed upside down on the top of a plastic cylinder surrounding a barberry seedling. This arrangement was kept in a dew chamber for 16 h in darkness, then placed in a greenhouse under the same conditions as for producing urediniospores. Ten days after basidiospore inoculation, pycnia could be observed. Pycniospores in nectar were harvested by being aspirated with a pipette and dried in a lyophilizer for 24 h before being stored at -80 °C. Pycniospores in nectar were transferred to different pycnia for fertilization to produce aeciospores. Ten days after fertilization, aeciospores were collected from barberry leaves using a sterile scalpel and kept at -80 °C.

For each spore type sample, three biological replicates were produced by harvesting spores from different plants grown in different pots or from leaf segments in different petri dishes. The morphological characteristics of the five different types of spores were observed by scanning electron microscope. The samples were cut into 0.5 X 0.5 cm pieces and incubated at 4 °C for 4—5 h in 4% glutaraldehyde with 0.2 M phosphate buffer, pH 6.8. Samples were washed four times with phosphate buffer, and gradually dehydrated with ethanol at 30%, 50%, 70%, 80%, 90%, 15 min for each concentration and three times with 100% ethanol for 30 min each. isoamyl acetate was used to treat the samples twice for 20 min. All the samples surface were collected with no touching and treatment within a minute. Sample surfaces were sputter-coated with gold in an E-1045 (Hitachi, Japan) after drying in CO_2_ vacuum and observed with an S-4800 SEM (Hitachi, Japan). All samples were kept at -80 °C for later nucleic acid isolation. Total RNA was extracted from 20 mg samples of urediniospores, teliospores, basidiospores, aeciospores, and pycniospores, respectively. RNA extraction was performed using a RNeasy Mini Kit (Qiagen) and genomic DNA was removed by in-column DNase treatment following the manufacturer’s protocol. RNA integrity was confirmed by 1.2% agarose gel electrophoresis and the concentration was determined using NanoDropTM 1000 (Thermo Fisher Scientific, Waltham, MA, USA).

### Transcriptome sequencing

A total of 1 μg RNA per sample was used for RNA sequencing library preparation. Sequencing libraries were generated using NEBNext® Ultra™ Directional RNA Library Prep Kit for Illumina® (New England Biolabs, Ipswich, ME, USA) following the manufacturer’s recommendations. Total mRNA was purified using poly-T oligo-attached magnetic beads. Fragmentation was carried out using divalent cations under elevated temperature in the NEBNext First Strand Synthesis Reaction Buffer (5X). First strand cDNA was synthesized using random hexamer primer and M-MuLV Reverse Transcriptase (RNaseH free), and second strand cDNA synthesis was subsequently performed with DNA Polymerase I and RNase H. Before sequencing, library quality was assessed on the Agilent Bioanalyzer 2100 system, then 150 bp paired-end reads were sequenced using the Illumina Hi-Seq 2000 Platform (San Diego, CA, USA).

### Gene expression analysis

In-house perl scripts were used to carry out adapter and barcode trimming as well as quality filtering. In this step, clean data (clean reads) were obtained by removing reads containing adapter, reads containing ploy-N and low quality reads from raw data. At the same time, Q20, Q30 and GC content the clean data were calculated. Clean paired-end reads were aligned to the genome of CY32 (Zheng et al. 2013) (Accession number CNSA: CNP0001524, https://db.cngb.org/cnsa/) using Hisat2 v2.0.4 with default parameters (Kim et al. 2015). HTSeq v0.9.1 was used to count the reads mapped to each gene (Anders et al. 2015). Fragments per kilobase of exon per million fragments mapped (FPKM) for each gene was calculated based on the length of the gene and read counts mapping to the gene. Differential expression analysis between two treatments was performed using the DESeq R package (1.18.0) (Wang et al. 2010), and identified genes with an adjusted *P*-value < 0.05 were considered as differentially expressed. The clustering analysis were used all the differentially expressed genes by the pheatmap R package (1.0.12). Principal component analyses, Pearson correlations between samples, and heatmaps of gene expression profiles were generated using software R 3.4.0.

### GO enrichment analysis of differentially expressed genes

Gene ontology (GO) enrichment analysis of differentially expressed genes was conducted using the GOseq R package, in which gene length biases were corrected (Young et al. 2010). GO terms with corrected *P* values less than 0.05 were considered to be significantly enriched.

### Alternative splicing (AS) analysis

We utilized rMATS (v4.0.1) (Shen et al. 2014) for AS analysis, whereby short reads were used to compare differences in FPKM in specific regions of the transcripts derived from each gene. For each AS event, rMATS calculated the percentage of exon inclusion (IncLevel) for each sample across the biological triplicates and detected differential IncLevel (IncLevelDifference) between different spores. The AS events were corrected by *P* values and false discovery rate (FDR) less than 0.05. The splicing event categories included skipped exon (SE), alternative 5' splice site (A5SS), alternative 3' splice site (A3SS), mutually exclusive exon (MXE), and retained intron (RI). Plots of AS events were generated using rmats2sashimiplot (v2.0.3), and heatmaps were generated by TBtools (Chen et al. 2020).

## Supplementary Information

**Additional file 1: Supplementary Table 1.** Number of RNA-seq reads and mapped reads in different spore stages of *Puccinia striiformis* f. sp. *tritici*.

**Additional file 2: Supplementary Table 2.** Significantly enriched gene functions of commonly expressed genes in all spore stages.

**Additional file 3: Supplementary Table 3.** Enriched GO terms for genes specifically expressed in basidial and pycnial stages.

**Additional file 4: Supplementary Table 4.** Relative expression level of *Puccinia striiformis* f. sp. *tritici* genes in different spore stages. Gene ID and expression values (FPKM) of DEGs in at least one stage compared to another with *p* <0.05.

**Additional file 5: Supplementary Table 5.** GO terms of *Puccinia striiformis* f. sp. *tritici* genes expressed in different spore stages organized by clusters.

**Additional file 6: Supplementary Table 6.** GO functional annotation of up-regulated genes in basidiospores, pycniospores, teliospores, and aeciospores when compared with genes in urediniospores.

**Additional file 7: Supplementary Table 7.** Genes regulated in basidiospores with a minimum of ten-fold change when compared with genes in urediniospores.

**Additional file 8: Supplementary Table 8.** Genes regulated in pycniospores with a minimum of ten-fold changes when compared with genes in urediniospores.

**Additional file 9: Supplementary Table 9.** Genes regulated in teliospores with a minimum of ten-fold changes when compared with genes in urediniospores.

**Additional file 10: Supplementary Table 10.** Alternative splicing events list.

## Data Availability

All data and materials are available in the paper and online supplemental files.
